# Supplementary material for: Stabilization of F-Actin Cytoskeleton by Paclitaxel Improves the Blastocyst Developmental Competence through P38 MAPK Activity in Porcine Embryos
Source: Biomedicines. 2022 Aug 2;10(8):1867. doi: 10.3390/biomedicines10081867 (PMC9405004; doi:10.3390/biomedicines10081867)
Supplement: Supplementary file 1 [file biomedicines-10-01867-s001.zip › Supplementary Figure S1..pptx]

## Slide 1
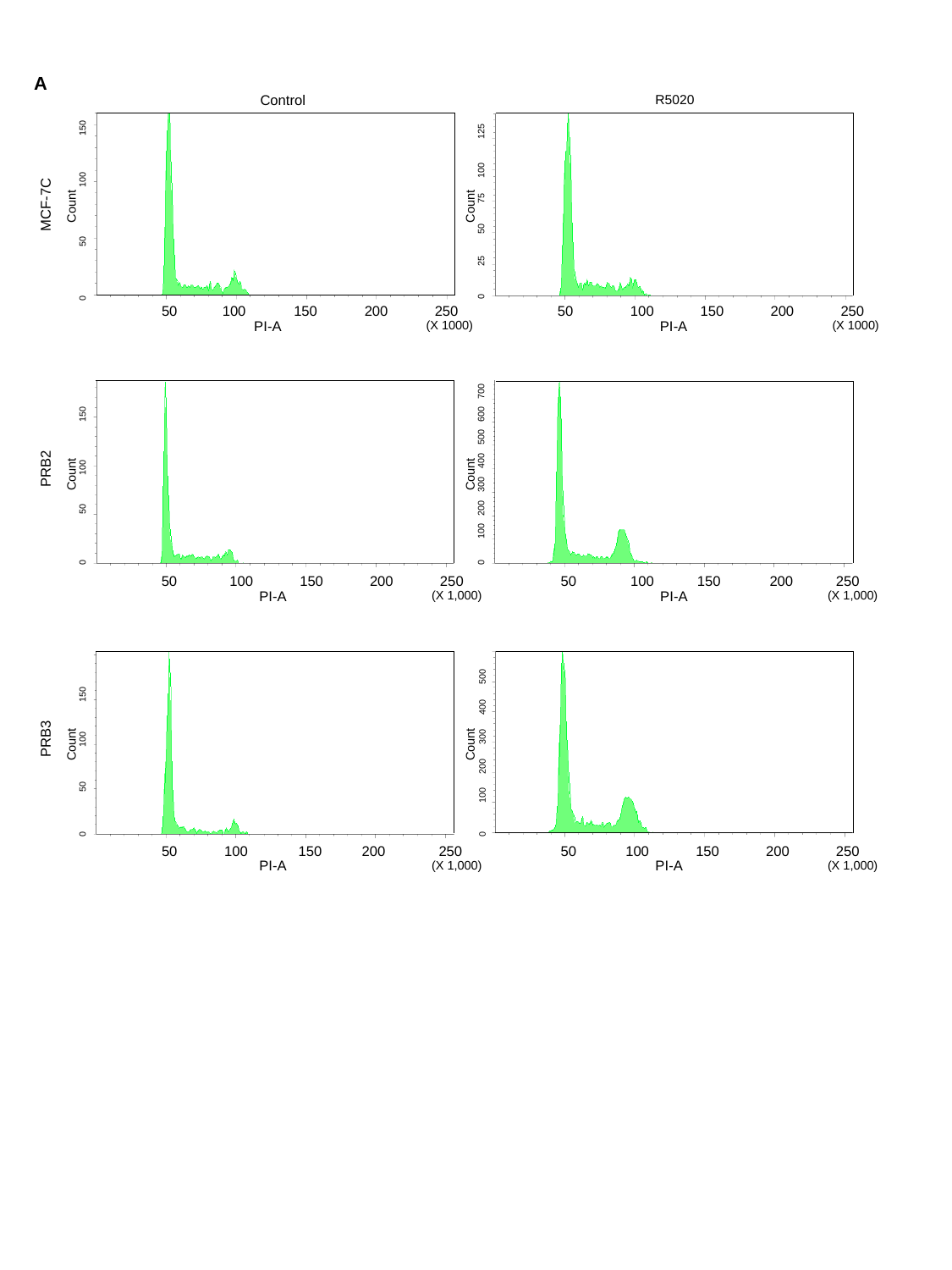

A
Control
150
100
50
0
Count
R5020
125
100
Count
75
50
25
0
MCF-7C
50
100
150
200
250
(X 1000)
PI-A
50
100
150
200
250
(X 1000)
PI-A
150
100
50
0
Count
PRB2
700
600
500
400
300
200
100
0
Count
50
100
150
200
250
(X 1,000)
PI-A
50
100
150
200
250
(X 1,000)
PI-A
150
100
50
0
Count
PRB3
500
400
300
200
100
0
Count
50
100
150
200
250
(X 1,000)
PI-A
50
100
150
200
250
(X 1,000)
PI-A

## Slide 2
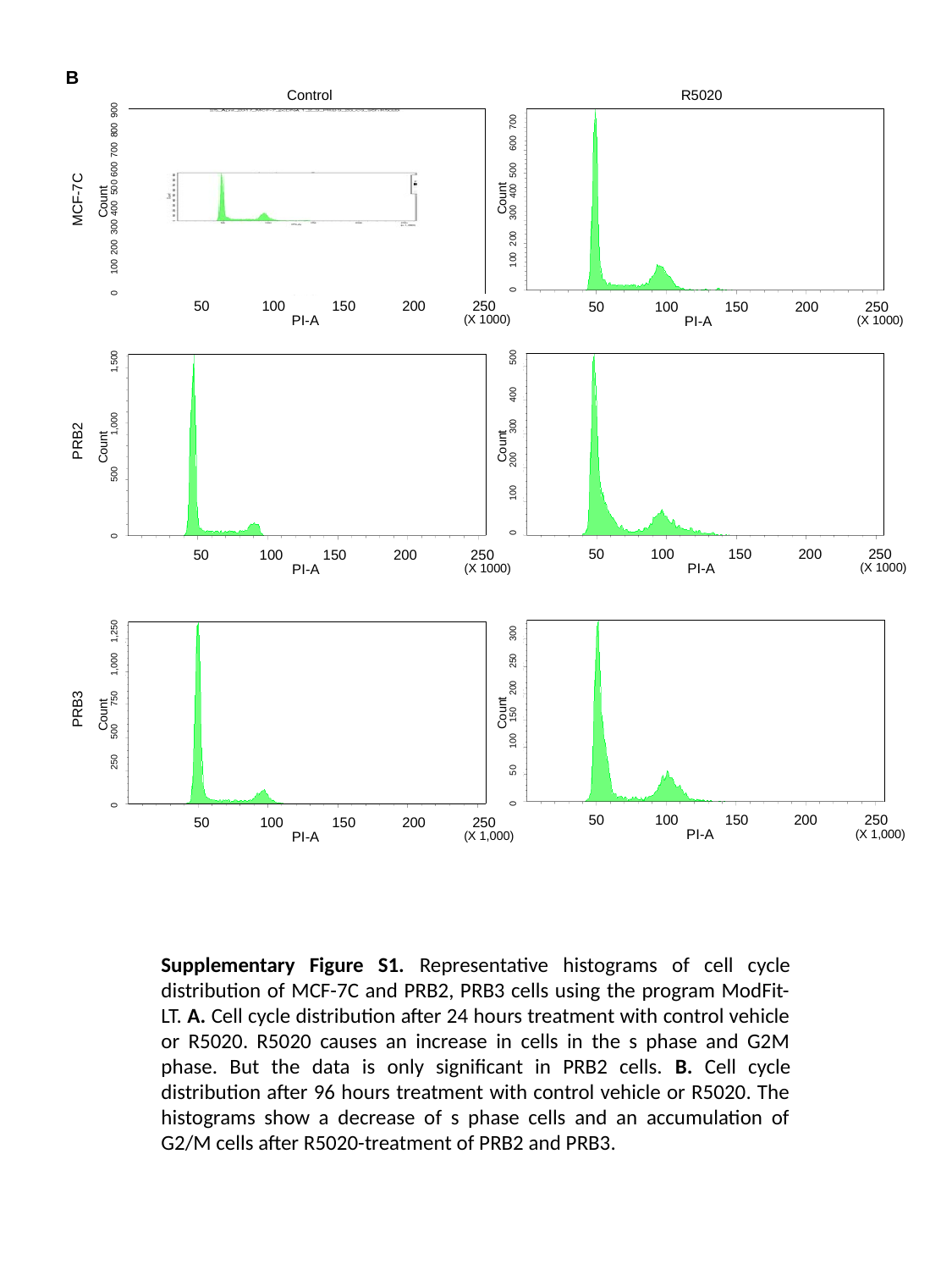

B
R5020
Control
900
800
700
600
500
400
300
200
100
0
Count
700
600
500
400
300
200
100
0
Count
MCF-7C
50
100
150
200
250
(X 1000)
PI-A
50
100
150
200
250
(X 1000)
PI-A
1,500
1,000
500
0
Count
PRB2
500
400
300
200
100
0
Count
50
100
150
200
250
(X 1000)
PI-A
50
100
150
200
250
(X 1000)
PI-A
1,250
1,000
750
500
0
Count
PRB3
250
300
250
200
150
100
50
0
Count
50
100
150
200
250
(X 1,000)
PI-A
50
100
150
200
250
(X 1,000)
PI-A
Supplementary Figure S1. Representative histograms of cell cycle distribution of MCF-7C and PRB2, PRB3 cells using the program ModFit-LT. A. Cell cycle distribution after 24 hours treatment with control vehicle or R5020. R5020 causes an increase in cells in the s phase and G2M phase. But the data is only significant in PRB2 cells. B. Cell cycle distribution after 96 hours treatment with control vehicle or R5020. The histograms show a decrease of s phase cells and an accumulation of G2/M cells after R5020-treatment of PRB2 and PRB3.
